# Multiphysics Modeling of Heat Transfer and Melt Pool Thermo-Fluid Dynamics in Laser-Based Powder Bed Fusion of Metals

**DOI:** 10.3390/ma18133183

**Published:** 2025-07-05

**Authors:** Tingzhong Zhang, Xijian Lin, Yanwen Qin, Dehua Zhu, Jing Wang, Chengguang Zhang, Yuchao Bai

**Affiliations:** 1 School of Mechanical and Electrical Engineering, Zhoukou Normal University, Zhoukou 466001, China; zhangchengguang@126.com; 2 College of Mechanical and Electrical Engineering, Wenzhou University, Wenzhou 325035, China; linxijian220@163.com (X.L.); zhu_556@163.com (D.Z.); 3 Shanxi Center of Technology Innovation for Light Manipulations and Applications, School of Applied Science, Taiyuan University of Science and Technology, Taiyuan 030024, China; qinyanwen4694@163.com (Y.Q.); wangjingtyust@163.com (J.W.); 4 School of Robotics and Advanced Manufacture, Harbin Institute of Technology, Shenzhen 518055, China

**Keywords:** laser-based powder bed fusion of metals, heat transfer, melt pool, level-set method, thermo-fluid dynamics

## Abstract

Laser-based powder bed fusion of metals (PBF-LB/M) is one of the most promising additive manufacturing technologies to fabricate complex-structured metal parts. However, its corresponding applications have been limited by technical bottlenecks and increasingly strict industrial requirements. Process optimization, a scientific issue, urgently needs to be solved. In this paper, a three-phase transient model based on the level-set method is established to examine the heat transfer and melt pool behavior in PBF-LB/M. Surface tension, the Marangoni effect, and recoil pressure are implemented in the model, and evaporation-induced mass and thermal loss are fully considered in the computing element. The results show that the surface roughness and density of metal parts induced by heat transfer and melt pool behavior are closely related to process parameters such as laser power, layer thickness, scanning speed, etc. When the volumetric energy density is low, the insufficient fusion of metal particles leads to pore defects. When the line energy density is high, the melt track is smooth with low porosity, resulting in the high density of the products. Additionally, the partial melting of powder particles at the beginning and end of the melting track usually contributes to pore formation. These findings provide valuable insights for improving the quality and reliability of metal additive manufacturing.

## 1. Introduction

Additive manufacturing (AM) differs from traditional subtractive manufacturing, such as drilling, cutting, casting, forging, welding, heat treatment, etc. [1], as it is an innovative technology that, through computer control, can fabricate complex parts such as internal cavities, topologically optimized structures, and integrated parts as designed. Moreover, it offers advantages including, but not limited to, high material utilization rate, light weighting, performance optimization, customization, and multi-functional integration [2]. In recent years, the market share of AM technology and the number of published papers have experienced exponential growth [3]. As one of the important AM technologies, the laser-based powder bed fusion of metals (PBF-LB/M) is becoming commercialized for engineering metal fabrication [4]. Currently, PBF-LB/M is being increasingly used in aerospace, automotive, and bio-implant industries, etc. [5]. In the process of PBF-LB/M, the laid metal particles irradiated by the laser beam absorb energy and then experience physical phenomena such as temperature rise, melting, evaporating, etc. Finally, the melt pool is formed. Under the evaporation-induced recoil pressure, the fluid in the melt pool spreads out and then infiltrates into the previous layer; as the laser beam moves away, the melt materials solidify and combine with each other, forming a track. Thus, track by track, layer by layer, the metal component is achieved.

**Figure 1:**
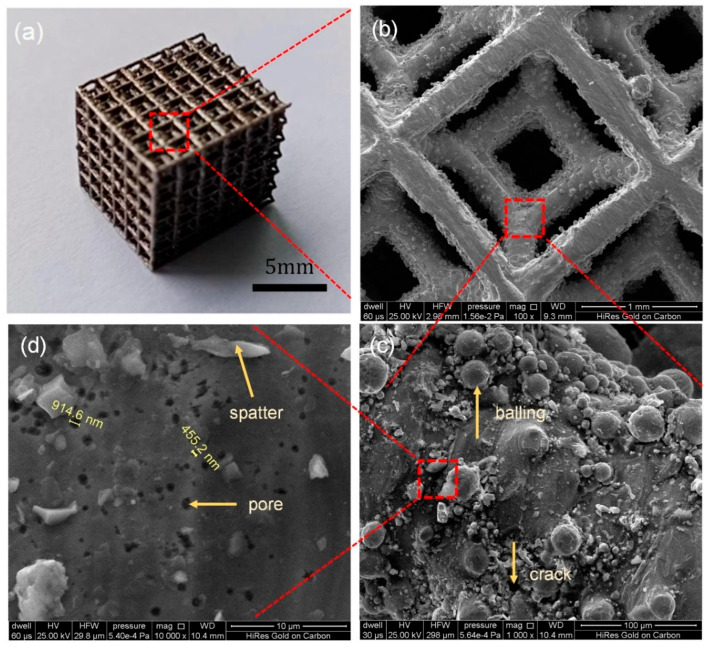
The defects of spatter, porosity, balling, etc. occurring in the PBF-LB/M process: (**a**) porous sample, (**b**) corresponding magnified image, (**c**) balling defect and (**d**) spatter and pore defects.

Although the basic concept of PBF-LB/M seems simple, the actual manufacturing process involves intricate multi-physics interactions. These include powder bed consolidation dynamics, thermal energy transfer mechanisms, metallurgical phase transitions, and melt pool thermo-fluid dynamics, all of which significantly shape both process stability and final product quality. Studies have shown that unstable melt pool dynamics can lead to various quality issues, including spatter [6,7,8], porosity [9,10], and balling effects [11,12,13,14]. These defects, illustrated in Figure 1, critically degrade mechanical performance and corrosion resistance in the finished metal parts, behavior which was confirmed in our experiments. Defect formation is due to both powder characteristics (size distribution, morphology) and laser parameters (power, scan strategy). In principle, heat transfer and melt pool thermo-fluid dynamics in PBF-LB/M play a vital role in quality control, which attracts substantial academic and industrial research. Both experimental and modeling approaches are being actively pursued to better understand these phenomena.

Modern experimental techniques have realized direct observation of the fundamental physical phenomena governing metal powder melting and solidification dynamics during the PBF-LB/M process [15,16]. In a pioneering study, King et al. [17] first revealed the keyhole effect and the pore formation mechanism in PBF-LB/M through high-speed X-ray imaging. Later, Zhao et al. [18] employed synchronous X-ray imaging and diffraction techniques to analyze the dynamic process and phase transformation of the melt pool. Hooper et al. [19] measured the temperature field of the melt pool and its cooling rate using a dual-wavelength, high-temperature meter. Leung et al. [20] revealed pore formation, solidification cracking, and melt flow in real time via synchrotron X-ray imaging in the PBF-LB/M process and proposed a pore bursting mechanism. Mohammad et al. [21] and Guo et al. [22] realized observation of the melt pool in situ using X-ray imaging and revealed the thermo-fluid dynamics of the melt pool via tracing particles. Cunningham et al. [23] defined the keyhole formation threshold and viewed the morphology evolution process through ultra-high transmission X-ray imaging. As artificial intelligence technology has progressed, Zhang et al. [24] have recently predicted the behavior of the melt pool based on the physical information neural network and the operando observation technique.

Experimental surveys have provided valuable empirical data for analyzing the heat transfer and thermo-fluid properties of the melt pool and offered conventional parameter optimization approaches. However, trial-and-error experiments have been proven to be both resource-intensive and inefficient, indicating that such methods are unsatisfactory within the limitations of resolution in terms of time and space.

Computational modeling has emerged as a powerful complementary approach, offering unique capabilities to reveal the complex multi-physics interactions during PBF-LB/M. Researchers have developed various computational frameworks to elucidate the complex physical phenomena governing thermo-fluid behavior [25,26]. For example, Zacharia et al. [27] carried out pioneering work and first applied the thermo-fluid coupling model to simulate the welding melt pool, laying the foundation for the subsequent PBF-LB/M model. Körner et al. [28] presented a 2D lattice Boltzmann model to study melting and re-solidification in randomly packed powder beds during the PBF-LB/M process, which provides new insights into melt pool dynamics. Among the various models, computational fluid dynamics (CFD) is the method widely used to simulate melt pool behavior [29]. Cao et al. [30] established a hydrodynamic model based on Fluent software and calculated and analyzed the solidified track dimensions in the Inconel 718 alloy PBF-LB/M process. Later, Cao et al. [31] analyzed the influences of scanning strategies on grain orientation, pore defects, and surface roughness based on the open-source finite volume method framework, OpenFOAM. However, in their models, the gas phase, which is crucial for understanding the PBF-LB/M, is lacking.

Some researchers have commonly applied DEM simulations for powder spreading processes, importing the size and position of the resulting powder bed into CFD models to analyze melt pool behavior. Khairallahn et al. [32], employing the multi-physics field model (coupling of heat, fluid, phase change, and stress) of ALE3D, studied melt pool behaviors and thus, the formation of pores, spatter, and denudation zone defects. Long et al. [33] studied a multi-layer PBF-LB/M process, depicting the melt pool characteristics and thermal behavior, and then analyzed the effects of scanning strategy and hatch spacing on the surface roughness and porosity of the parts. Zhang et al. [34] developed a CFD-DEM-CALPHAD coupling model to simultaneously reveal the gas, melt pool, and particle dynamics in single- and multi-material PBF-LB/M processes. They found that the inconsistency of the melt track is caused by the powder spattering and entrainment in the PBF-LB/M process, and the large agglomeration induced by the hot spatter coalescence can cause lack-of-fusion and porosity defects. Yu et al. [35] conducted CFD and DEM simulations to capture vapor-driven keyhole dynamics, melt pool morphology evolution, and powder motions. Bayat et al. [36] used the Flow-3D commercial software to analyze the formation, evolution, and disappearance of keyhole and keyhole-induced porosities during the Ti6Al4V alloy single-track PBF-LB/M process. The single-track PBF-LB/M process was numerically examined by Wu et al. [37], who used the finite volume method (FVM), with particular focus on evaporation effects. It was found that when evaporation was neglected, the characteristics of the melt pool were not accurately reflected, yielding excessively and unrealistically high temperatures. The above-mentioned models are usually accompanied by poor convergence in multiple software integration calculations, and in some cases, the mass loss caused by evaporation is ignored, resulting in the inaccurate calculation of the melt pool thermo-fluid dynamics property.

In this paper, we establish a comprehensive three-phase transient model based on the level-set method to investigate heat transfer and melt pool dynamics in PBF-LB/M. In the model, the finite element method is employed using COMSOL 6.3 version Multiphysics software, incorporating key physical phenomena including surface tension, Marangoni convection, and recoil pressure. The model accounts for both thermal dissipation and mass losses due to evaporation, as well as the interaction between the laser beam and powder bed, where the laser is modeled as a surface heat flux acting on the powder particles.

## 2. Model Description

**Figure 2:**
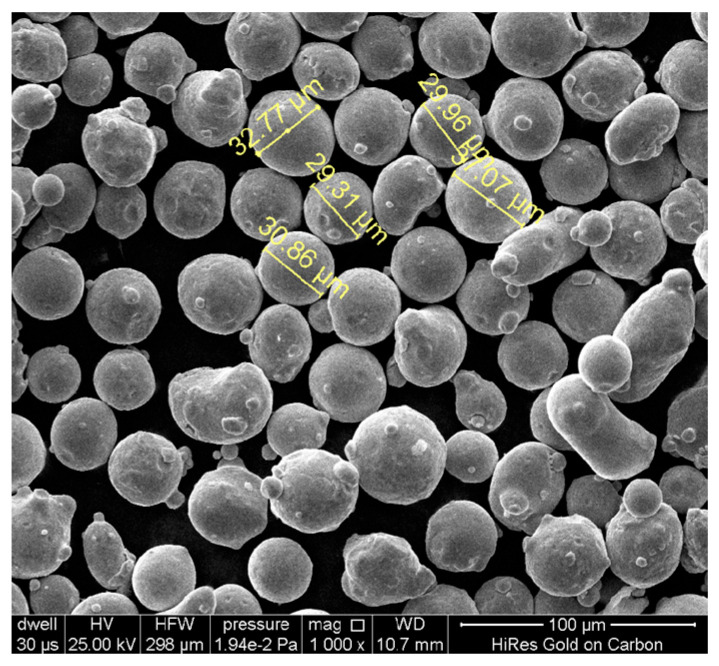
Characteristics of powders observed using scanning electron microscopy.

**Figure 3:**
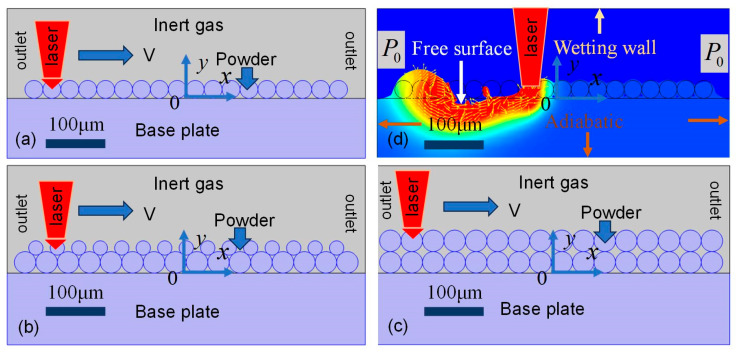
PBF-LB/M model displaying (**a**) single-layer powder thickness, (**b**) single-layer powder accompanied by satellite clusters, (**c**) double-layer powder thickness, and (**d**) schematic diagram.

In the experiments, the metal particles are Ti-6Al-4V, the geometric characteristics of which are presented in Figure 2. From the data obtained via scanning electron microscopy, the powder diameter measures about 30 μm. Meanwhile, some powders often exhibit a satellite powder cluster phenomena. Considering the influence of the powder thickness on the metal part, three models are established by varying the powder thickness, as shown in Figure 3.

Based on the metal powder property, the computational model is constructed with dimensions of 600 μm in length and 250 μm in height. The computation region is composed of two distinct regions: a 100 μm thick solid substrate at the bottom and a 150 μm gas domain above it. The four critical process parameters governing the PBF-LB/M process include (1) laser power, (2) beam spot radius, (3) scanning velocity, and (4) powder layer thickness. These parameters collectively define the thermal-fluid phenomena during the PBF-LB/M process, which is presented in Table 1.

The structural diagram of PBF-LB/M is shown in Figure 3. As illustrated in Figure 3a, a monolayer of uniformly sized 30 μm particles is arranged in an ordered packing configuration, consisting of 18 spherical powders on the base plate. Figure 3b introduces a secondary finer particle, with 15 satellite powders (15 μm diameter) positioned at the junctions between the primary particles. Figure 3c extends this configuration to a double-layer packing scheme using the same 30 μm particles. The powder material properties of Ti-6Al-4V used in this work are listed in Table 2.

### 2.1. Simulation Conditions

In the numerical model, consistent material properties are employed for both the substrate and powder bed, with argon serving as the shielding gas. The thermal boundary conditions are specified as follows: 300 K is set as the initial temperature for the whole commutation region; adiabatic conditions are set on the base plate outside three sides. Hydrodynamic boundary conditions: A zero-pressure gradient boundary is set for free surface (gas–powder interface) treatment. The pressure boundary in the gas region at the outlet is $p=p_{0}$, and the top side is a wetting wall; in the solid region, three sides are zero slip types.

The laser source is modeled as a Gaussian distribution acting on the free surface, with an initial beam position $x_{0}=-260\;\mu m$ and unidirectional scanning along the x-axis. This configuration enables the simulation of single-track PBF-LB/M, while maintaining high computational efficiency through appropriate boundary condition simplifications.

### 2.2. Mathematical Model

In the present study, in the schematic of the physical model for PBF-LB/M shown in Figure 3, the following assumptions are made.

(1) The fluid flow is Newtonian, laminar, and incompressible.
(2) The vapor plume, comprising a metallic gas and an inert gas, is an ideal gas that is transparent to the incident laser beam.
(3) The Boussinesq approximation is implemented in the numerical model to address thermal-induced density variations in the melt pool.
(4) The enthalpy-porosity technique is adopted to simulate the metal powder melting and re-solidification process.

Based on these assumptions, a three-phase transient model coupling the finite element method, the level-set technique, and hydrodynamic equations is established for simulating the heat transfer and thermo-fluid dynamics in PBF-LB/M. The mathematical model is illustrated by modified continuity Equation (1), Navier–Stokes Equation (2), energy conservation Equation (3), and level-set Equation (4).

(1) $$\nabla \cdot \vec{U}=m_{mfr}\delta \left(\phi \right)\left(\frac{\rho_{l}-\rho }{\rho }\right)$$

(2) $$\frac{\partial \left(\rho \vec{U}\right)}{\partial t}+\vec{U}\cdot \nabla \left(\rho \vec{U}\right)=-\nabla p+\nabla \cdot \left(\mu \nabla \vec{U}\right)+\vec{F_{A}}+\vec{F_{B}}+\vec{F_{C}}\delta \left(\phi \right)$$

(3) $$\rho C_{p}[\frac{\partial T}{\partial t}+\nabla \cdot \left(\vec{U}T\right)]=\nabla \cdot \left(kT\right)+Q\delta \left(\phi \right)$$

(4) $$\frac{\partial \phi }{\partial t}+\vec{U}\cdot \nabla \phi -m_{mfr}\delta \left(\phi \right)\left(\frac{\phi }{\rho_{l}}+\frac{1-\phi }{\rho_{v}}\right)=\chi \nabla \cdot \left(\zeta \nabla \phi -\phi \left(1-\phi \right)\frac{\nabla \phi }{\left|\nabla \phi \right|}\right)$$

In Equation (1), $\delta \left(\phi \right)$, ϕ, ρ, $\rho_{l}$, and $\vec{U}$ are the delta function, level-set function, density, liquid phase density, and the flow velocity, respectively. The additional source term in the right equation in which the evaporation phenomenon is imported depends on the mass flow rate $m_{mfr}$, which is related to the local saturated vapor pressure $p_{s}\left(T\right)$ [40].

(5) $$m_{mfr}=\sqrt{\frac{m}{2\pi k_{b}}}\frac{p_{s}\left(T\right)}{\sqrt{T}}\left(1-\beta_{r}\right)$$

(6) $$p_{s}\left(T\right)=p_{0}\exp\left(L_{v}\frac{T-T_{v}}{k_{b}TT_{v}}\right)$$

Here, m is the atomic weight of the metal, $k_{b}$ is the Boltzmann constant, $p_{s}\left(T\right)$ is the saturated vapor pressure, $p_{0}$ is the atmosphere pressure, $T_{v}$ is the vaporization temperature, $L_{v}$ is the latent heat of vaporization, and *β*_r_ is the retro-diffusion coefficient, assumed to be equal to 18%. Admittedly, all the gas phases are assumed to be ideal gases and to obey the ideal gas law.

In Equation (2), μ is the viscosity. The additional source term, Darcy damping force $\vec{F_{A}}$, originating from the enthalpy-porosity technique, is incorporated in the numerical model as a damping mechanism to accurately represent the gradual velocity reduction occurring in the mushy zone during solid–liquid phase transition. This treatment accounts for the increasing flow resistance as the material transforms from a liquid to a solid state.

(7) $$\vec{F_{A}}=-[\frac{K_{0}{\left(1-g_{l}\right)}^{2}}{g_{l}^{3}+e_{o}}\frac{\rho }{\rho_{L}}]\vec{\cdot U}$$

Here, *K*_0_ is the drag coefficient for a porous media model derived from the enthalpy-porosity technique, i.e., 6 × 10^4^ kg/(m^3·s); $e_{0}$ is a very small value (0.001), avoiding a zero denominator; and $g_{l}$ is the liquid volume fraction. The Boμssinesq approximation is formulated to account for the density change caused by temperature variation in the melt pool, described as $\vec{F_{B}}$.

(8) $$\vec{F_{B}}=\rho \vec{g}-\rho \vec{g}\beta_{l}\left(T-T_{m}\right)$$

Here, *β*_l_ is the thermal expansion coefficient, $T_{m}$ is the melting point, and *T* is the real temperature.

Another additional source term, acting on the free surface, is expressed as $\vec{F_{C}}$.

(9) $$\vec{F_{C}}=\gamma \vec{n}\kappa -\nabla_{\vec{s}}T\frac{\partial \sigma }{\partial T}+P_{R}\left(T\right)\vec{n}$$

Here, $\vec{n}$ and $\vec{s}$ are the free surface normal vector and tangential vector, respectively. κ is the face curvature, which is expressed as κ=−[∇⋅n→n]; ∂σ/∂T is the surface tension gradient, and $\nabla_{\vec{s}}T$ is temperature gradient; $P_{R}\left(T\right)=0.54p_{s}\left(T\right)$ is the recoil force.

Thus, surface tension, the Marangoni effect, and the recoil force coupled in $\vec{F_{C}}$ are all incorporated into the governing equation acting on the free surface, making the calculation more accurate.

In Equation (3), k is the thermal conductivity; $C_{p}$ is the equivalent specific heat capacity. In order to deal with the latent heat caused by phase transitions, the equivalent specific heat capacity method is used in the numerical simulation model, which is expressed in the following Equation (10).

(10) $$C_{p}=C_{p0}+L_{m}\exp\left(-{\left(T-T_{m}\right)}^{2}/dT_{m}\right)/\sqrt{\pi }dT_{m}+L_{v}\exp\left(-{\left(T-T_{v}\right)}^{2}/dT_{v}\right)/\sqrt{\pi }dT_{v}$$

Here, $C_{p0}$ is the initial specific heat capacity; $T_{m}$ and $T_{v}$ are the melting and evaporation points, respectively; $L_{m}$, $L_{v}$ is the latent heat of melting and evaporation, respectively; $dT_{m}$ and $dT_{v}$ are the transition temperatures across the melting or evaporation temperature, respectively.

In Equation (3), Q is the heat flux source, including irradiated laser energy and energy dissipation due to evaporation, convection, and irradiation on the gas–liquid free surface, which is described as follows:

(11) $$Q\left(x,t\right)=\frac{\alpha P}{\pi r_{0}^{2}}\exp\left(\frac{-2{\left(x-x_{0}-vt\right)}^{2}}{r_{0}^{2}}\right)-m_{mfr}L_{v}-h_{c}\left(T-T_{0}\right)-\varepsilon k_{b}\left(T^{4}-T_{0}^{4}\right)$$

The first term on the right-hand side of the equation, as an additional source term, represents a moving Gaussian-distributed laser source acting on the powder bed surface. Here, *P* is the laser power, $r_{0}$ denotes the radial distance from the beam center, and α is the absorptivity of the powder affected by the laser wavelength. x is the radial distance from the beam center, and $T_{0}$ is the ambient temperature; $h_{c}$ is the convective heat transfer coefficient, and ε is the emissivity.

In the modified level-set transport equation (Equation (4)), an additional source term $m_{mfr}\delta \left(\phi \right)\left(\frac{\phi }{\rho_{l}}+\frac{1-\phi }{\rho_{v}}\right)$, relating to mass loss due to evaporation, has been incorporated to ensure the precision of the melt pool calculation, where χ and ζ are two level-set parameters, which are 10 m/s, and 1.5 × 10^−5^ m, respectively. $\rho_{v}$ is the density of the metallic vapor. The level-set method incorporates an additional fluid volume fraction function ϕ to define the value between 0 and 1 quantitatively at each grid cell, describing the phase distribution, in which 0 represents a completely void cell, and 1 represents a fully occupied cell.

**Figure 4:**
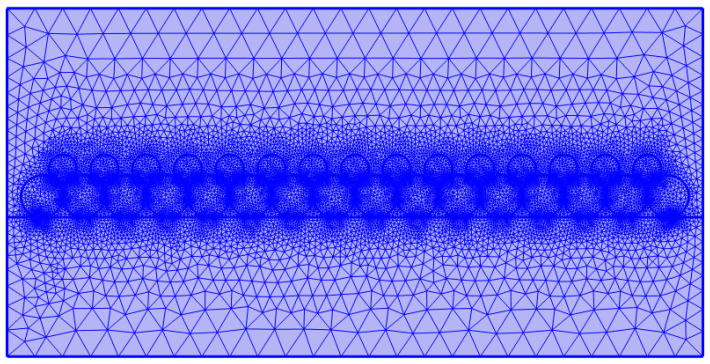
Mesh division of the computational domain.

At the beginning of the PBF-LB/M, the computation domain consists of the solid and gas regions. The heat source is transferred to the target as heat input, serving as the thermal boundary condition. At the free surface, the partial derivative is defined as follows: $-k\frac{\partial T}{\partial n}=Q\left(x,t\right)$. Equations (1)–(4) above are discretized and then solved with the finite element method in COMSOL Multiphysics. The computational domain employs a graded mesh system with progressive coarsening from the critical regions to the peripheral areas. Specifically, a fine mesh resolution of 10 μm is applied at the gas/particle/metal base interface to accurately resolve multiphase interactions; the adjacent region employs a maximum mesh size of 20 μm; the grid size gradually increases with the distance from the interface region to optimize computational efficiency while maintaining solution accuracy. The numerical solution utilizes a segregated solver approach with adaptive time stepping: a refined time step of 1 × 10^−6^ s during laser activation captures rapid thermal and fluid dynamics; an increased time step of 1 × 10^−5^ s during laser-off periods enhances computational efficiency. The complete mesh distribution and transition details are presented in Figure 4.

## 3. Results and Discussion

### 3.1. Melting and Solidification of Metal Powders and Model Validation

**Figure 5:**
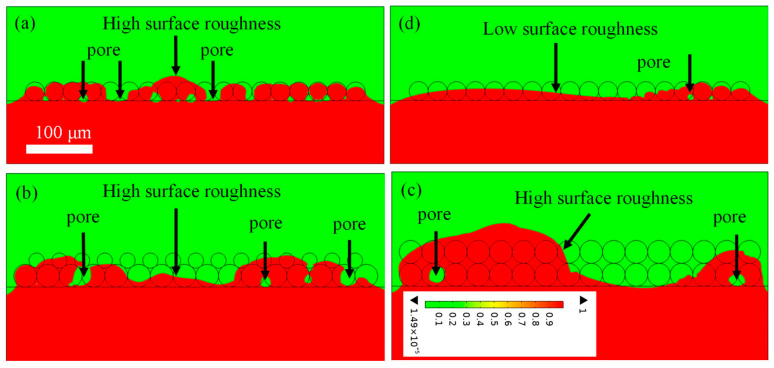
Simulation results of surface morphology of the melt track with (**a**) a single layer, laser power 130 w, scanning speed 2 m/s; (**b**) a single-layer accompanying a satellite layer, laser power 135 w, scanning speed 2 m/s; (**c**) a double-layer, laser power 175 w, scanning speed 2 m/s, and (**d**) a single-layer, laser power 200 w, scanning speed 1.8 m/s. (Red indicates metal, and cyan indicates gas.)

Figure 5 shows the simulation results of the surface morphology of the melt track in four different situations. The simulated solidified-surface morphology of the PBF-LB/M track is analyzed by measuring the surface heights at different locations, from which the surface roughness is determined, and the surface roughness Ra is calculated with standard deviation $\mathrm{Ra}=\sqrt{\sum_{i=1}^{N}\left(H_{i}-H_{a}\right)/N}$, where *H_i_* is the surface height at the measured point i, *H_a_* is the averaged surface height, and N is the total measured points (*N* = 12 in this work). The calculation results are (a) 11.5 μm; (b) 12.9 μm; (b) 29 μm; (d) 8.9 μm.

The surface roughness is related to the velocity, layer thickness, and laser power density. Here, the volume energy density $E_{\lambda }=\frac{P}{vDH}$ is defined to reveal the relationship between them. The calculation result is obtained for case (a–d) as follows: 6.6 × 10^10^ J/m^3^, 6.4 × 10^10^ J/m^3^, 4.8 × 10^10^ J/m^3^, and 1.2 × 10^11^ J/m^3^, respectively. The lower laser volume energy density results in a larger surface roughness. The higher the laser volume energy density, the lower the surface roughness. A comparison between the simulation and the experiment in Ref. [41] shows that the surface morphologies are inversely related to the laser volume energy density. This also confirms that the simulation system can be used to reveal the underlying physics behind the experiment.

**Figure 6:**
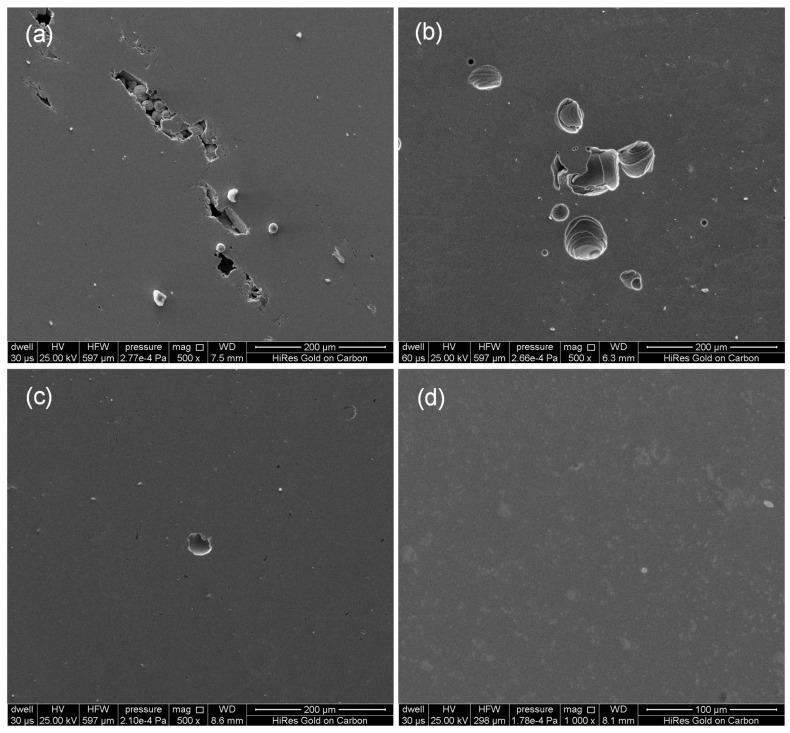
SEM results for PBF-LB/M-fabricated sample cross-sections under different processing parameters: (**a**) a single layer, laser power 130 W, scanning speed 2 m/s; (**b**) a single-layer accompanying a satellite layer, laser power 135 W, scanning speed 2 m/s; (**c**) a double-layer, laser power 175 W, scanning speed 2 m/s, and (**d**) a single-layer, laser power 200 W, scanning speed 1.8 m/s.

Additionally, the porosity is also calculated from Image J (Version 1.54p), which is a free image processing software based on Java. The obtained results are (a) 2.8%; (b) 2.9%; (c) 1%; (d) 0.1%. The corresponding experiments are conducted, and the sample is handled via wire cutting, polishing, and high-resolution scanning electron microscopy (SEM) observation. The SEM analysis was performed using an FEI Quanta 200 instrument (FEI Ltd. Guang zhou, China). This system offers a secondary electron resolution of 0.6 nm at 15 kV and 1.0 nm at 1 kV. The corresponding experimental data are presented in Figure 6. Via Image J software, the sample porosity is obtained as (a) 2.9%, (b) 2.98%, (c) 0.32%, and (d) 0.11%. Through comparative analysis, the experimental data are consistent with the simulation results, which verifies that the model is qualified for dealing with heat transfer and fluid flow in PBF-LB/M. Here, another important physical quantity, the line energy density $E_{v}=\frac{P}{V}$, is defined, which is closely related to porosity, resulting in (a) 60 W/m/s, (b) 67.5 W/m/s, (c) 87.5 W/m/s, and (d) 111 W/m/s. According to the calculation data, the larger linear energy density results in the higher density and the lower porosity.

### 3.2. Effects of Laser Power and Porosity Formation Mechanism

**Figure 7:**
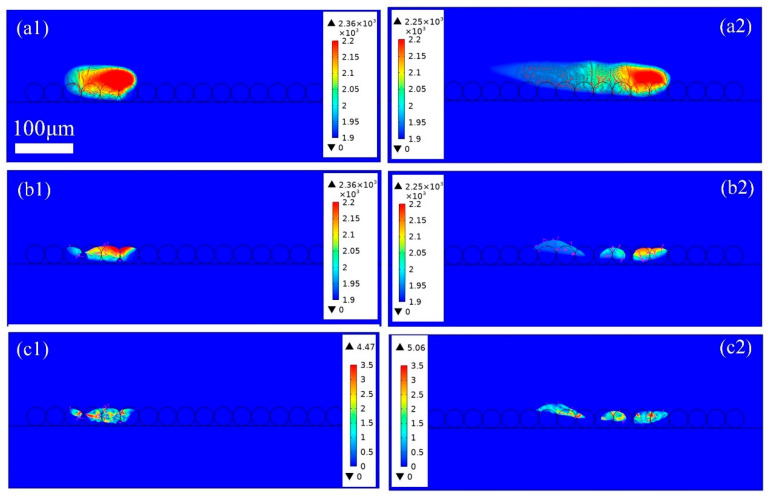
The thermo-fluid dynamics of (**a1**)100 μs and (**a2**) 240 μs the heat affected zone, (**b1**) 100 μs and (**b2**) 240 μs and (**c1**) 100 μs and (**c2**) 240 μs the melt pool in PBF-LB/M process with a single-layer particle, laser power 120 W, and scanning speed 2 m/s.

Figure 7 depicts the thermo-fluid dynamic properties across a single powder layer during PBF-LB/M processing, capturing the complete thermal history, including powder melting and molten pool formation at different time intervals. The laser beam scans from left to right, and powders are melted and even evaporated when the temperature reaches the melting and evaporation points. It should be noted that the temperature below the melting point is omitted in Figure 7, and only the melt pool and metal vapor are included in the model result. In Figure 7(a1), at 100 μs, the irradiated particle absorbs the laser energy, and the temperature rises to the melting point, eventually reaching the evaporation point. As the laser moves forward, which can be seen at 240 μs in Figure 7(a2), the previously irradiated region cools to room temperature and solidifies due to the removal of the laser beam and the heat dissipation from evaporation, convection, and irradiation. And the shape of the high-temperature area scanned by the laser is similar to that of a trailing comet. In addition, the temperature gradient at the back of the melting track is smaller than that at the front region. In the absence of the metal vapor, the morphology of the melt pool is acquired and is displayed in Figure 7b,c. In Figure 7b, the metal particle is not fully melted, and the melt pool is not consistent with the intermittent liquidus column phenomena. This is mainly due to the fact that the gap between the powders is filled with inert gas. Therefore, the thermal conductivity is very low, and heat dissipation is difficult. Insufficient energy input will lead to the presence of unsintered particles, resulting in poor metallurgical bonding, as can be seen in Figure 5a.

To more clearly demonstrate the transient evolution of the melt pool, the thermo-fluid fields of the melt track are shown in sequence in Figure 7c. In Figure 7c, the amount of fluid is not enough, and the melt pool is interrupted. In addition, the incomplete melting leads to a large dissipation force, which prevents the free mobility of the fluid. Additionally, due to the principle of surface minimum energy, the surface tension drives the fluid to contract and keeps it from spreading out. All these conditions further limit the spread of the liquid, forming independent liquid protrusions with uneven surfaces, which is confirmed in Figure 6a. Moreover, the maximum velocity of the fluid is only 3.8 m/s, and the average fluid is about 2 m/s. The flow is slow, with a small inertia force, and it cannot overcome the surface tension. The depression between particles is not filled by melt liquid in time, thus forming pores. Therefore, incomplete fusion is the main reason for the high surface roughness and high porosity. In addition, with the increase in the number of scans, the surface roughness and porosity will be further aggravated, which will reduce the mechanical properties and corrosion resistance of the metal part.

### 3.3. Effects of Layer Thickness and Porosity Formation Mechanism

**Figure 8:**
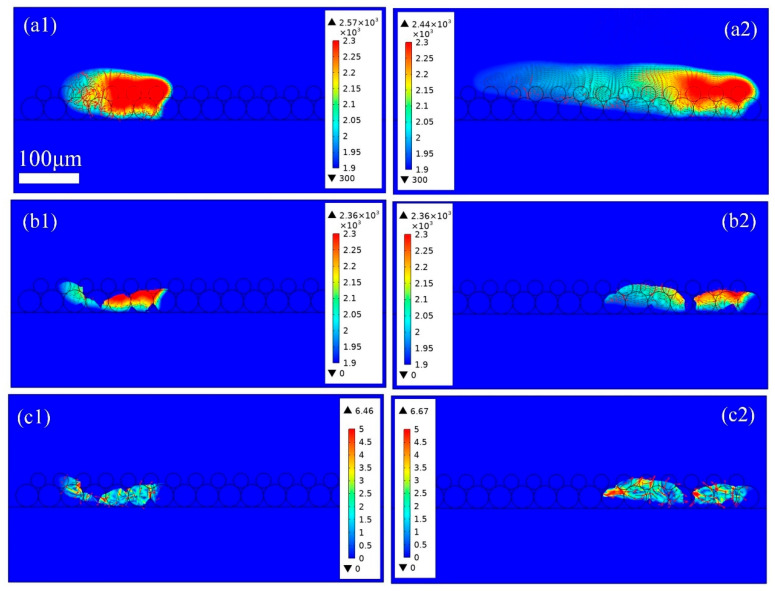
The thermo-fluid dynamics of (**a1**) 100 μs and (**a2**) 240 μs the heat affected zone; (**b1**) 100 μs and (**b2**) 240 μs and (**c1**) 100 μs and (**c2**) 240 μs the melt pool in PBF-LB/M process with layer thickness 34 μm, laser power 135 W, and scanning speed 2 m/s.

In the PBF-LB/M process, the layer thickness is another important parameter. In this section, the layer thickness is changed from 30 μm to 34 μm. Figure 8 shows the thermo-fluid properties along the track during the melting and solidification process with a layer thickness of 34 μm. With the increase in layer thickness, much more laser energy is needed to melt the powders, so the laser power is set to 135 W. In Figure 8a, the it can be seen that the evaporation phenomenon is severe, and the heat-affected area has significantly increased in the PBF-LB/M process. However, the comet-like outline of the high-temperature zone is roughly the same as that in Figure 7a, and will not be further investigated here. In Figure 8b, the satellite powder is fully melted and even half evaporated. However, the larger downside particles are only half melted, and the base plate is not melted at all. It can be concluded that, due to limited volume energy density input, incomplete melting is also serious. However, under the higher linear energy density input, more melt liquid emerges, thus forming a larger melt pool. In Figure 8c, it can be noted that the melt flow velocity has also increased. The enhanced flow enables a number of large-sized gas cavities, which are not completely released, to be trapped in the melt pool, thus forming pores, as confirmed in Figure 5b. The increase in layer thickness will lead to higher surface roughness and porosity, along with poorer metallurgical bonding with the base plate.

**Figure 9:**
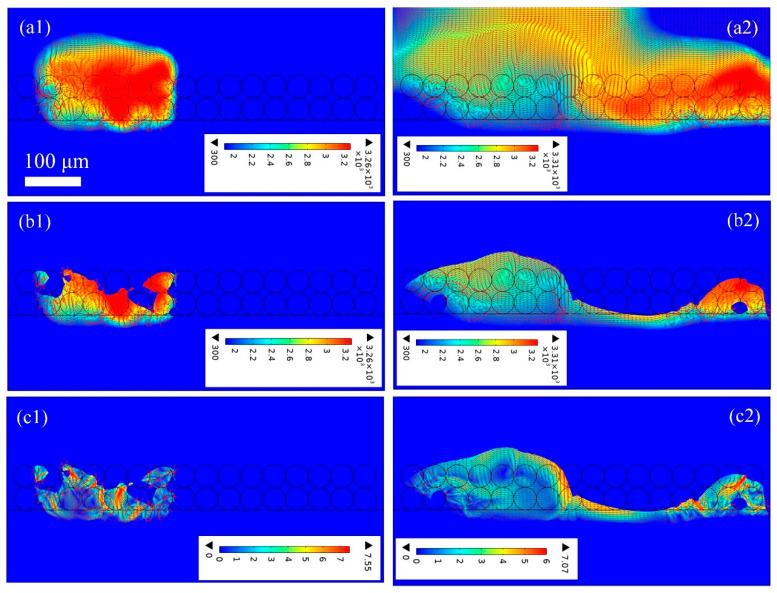
The thermo-fluid dynamics of (**a1**) 100 μs and (**a2**) 240 μs the heat affected zone; (**b1**) 100 μs and (**b2**) 240 μs and (**c1**) 100 μs and (**c2**) 240 μs the melt pool in PBF-LB/M process with layer thickness 60 μm, laser power 175 W, and scanning speed 2 m/s.

Figure 9 shows the thermo-fluid dynamics of the heat heat-affected zone in PBF-LB/M with a double layer thickness of 60 μm. In Figure 9a, the laser power is set to 200 W, and more metal powders are melted and evaporated, with the intense evaporation causing steam turbulence. The double-layer powder is completely melted, and some of the base plate also becomes melted. As the laser source scans in the +x direction to the right, the laser irradiated zone increases. However, the previous laser scanning zone has not completely cooled and solidified, resulting in the formation of a larger high-temperature area. In Figure 9(b1), it can be seen that melt pools with irregular shapes are formed with steep front walls as the laser source scans along the +x the direction at 240 μs, as shown in Figure 9(b2). The newly formed melt pool is connected with the previous melt pool, and thus a larger melt pool is formed. The shape of the melt pool is irregular, resembling the results of a duck playing in water. The continuity of the melt pool improves significantly at *P* = 200 W compared with the conditions at *P* = 120, 135 W. However, the rapid cooling and high surface tension at this energy density cause significant fluctuations, along with the formation of valleys along the melt track. As illustrated in Figure 9(b1,c1), most of the dissolved gas is released from the molten metal into the inert atmosphere. Nevertheless, due to limited laser energy input and thermal dissipation, such as conduction, convection, and evaporation, the powders at both ends of the melt track are only partially melted. In addition, due to wetting phenomena, gas within the powder bed is trapped in the melt liquid, leading to porosity at the start and end regions of the scanning track, as shown in Figure 9(b2,c2), which is consistent with the results shown in Figure 5c.

### 3.4. Process Parameter Optimization

**Figure 10:**
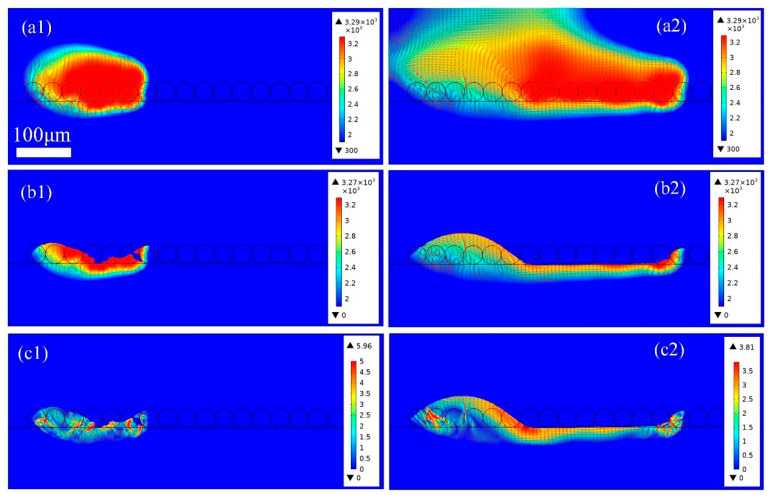
The thermo-fluid dynamics of (**a1**) 100 μs and (**a2**) 240 μs the heat affected zone; (**b1**) 100 μs and (**b2**) 240 μs and (**c1**) 100 μs and (**c2**) 240 μs the melt pool in PBF-LB/M process with layer thickness 30 μm, laser power 200 W, and scanning speed 1.8 m/s.

Figure 10 shows the thermo-fluid dynamics of the heat-affected zone in PBF-LB/M with a single layer thickness of 30 μm, a laser power of 200 W, and a scanning speed of 1.8 m/s. In Figure 10(a1), both the metal powders and the base plate are melted and evaporated under the higher laser energy input, and the evaporation is extremely intense, which can be seen in the red region. As the laser moves and the intense evaporation areas merge, a huge rainbow is formed, as seen in Figure 10(a2). Moreover, as the laser beam scans from left to right, the melt pool is enlarged due to the connection between the pre-melting pool and the current melting pool, which is mainly related to the thermo-fluid properties of the melt pool. The current high-temperature fluid moves to the left, adding the energy required for maintaining the temperature of the previous melt pool and preventing the premature solidification of the previous melt pool, which can be seen in Figure 10b. In addition, under the recoil pressure caused by strong evaporation, the fluid velocity increases, the fluidity also enhances, and the backward flow results in a smooth melt pool surface, which can be seen in Figure 10c. Then, the melt powder particles are fully integrated with the substrate, forming an indivisible melt pool. Upon solidification, a metal part with smooth surfaces and low porosity is formed. It should be noted that these conclusions can be confirmed by Ref. [7], which has reported that a depressed melt pool signifies stability, with a shallower depression contributing to improved surface quality and a higher bulk density of the solidified track.

## 4. Conclusions

In this study, a three-phase transient numerical model is developed to examine heat transfer and melt pool dynamics in the PBF-LB/M process. The model incorporates level-set methods coupled with modified mass, momentum, and energy conservation equations, and is rigorously validated against the experimental data. Parametric studies evaluate the influence of laser power, scanning speed, and powder layer thickness on melt pool behavior. The key findings are summarized as follows:

(1) With the lower volume energy density, the powder bed cannot be completely melted. Incomplete melting results in insufficient liquid spreading and limited liquid penetration, which causes higher porosity in the part due to insufficient liquid filling. Conversely, the higher volume energy density promotes gas dissolution, coalescence, and expulsion from the melt pool, with higher density in the part. Moreover, the porosity is usually exacerbated by rapid cooling rates, especially at the start and the end of the melt track.
(2) The lower line energy density causes the metal powder to melt incompletely, especially with insufficient evaporation. The incomplete melting leads to a large dissipative force, which limits the further flow of the fluid and causes the melt pools to separate from each other, which also limits heat transfer and liquid infiltration, resulting in a lower bonding strength and a larger surface roughness of the finished part. However, the higher line energy density can improve the rate of melting and evaporation, and thus, improved surface quality and higher density are achieved.

## Figures and Tables

**Table 1:** Laser heat source model and process parameters in the simulation.

Parameter (Symbol/Unit)	Value
Laser power (P/W)	120, 135, 175, 200
Laser beam radiμs (r_0_/μm)	35
Laser scan speed (v/m/s)	1.8, 2
Powder thickens (H/μm)	30, 34, 60
Particle diameter (D/μm)	30, 15

**Table 2:** Thermophysical material properties of Ti-6Al-4V alloy for PBF-LB/M [38,39].

Nomenclature	Value	Nomenclature	Value
Liquid Density ($\rho_{l}$)	4000 kg/m^3^	Melting Temperature (*T*_m_)	1923 K
Gas Density ($\rho_{v}$)	39.95 × 10^−3^ kg/mol	Boiling Temperature (*T*_v_)	3315 K
Viscosity (μ)	5 × 10^−3^ Pa s	Boltzmann Constant (*k*_b_)	1.38 × 10^−23^ J/K
Specific Heat ($C_{p0}$)	610 J/(kg/K)	Level-Set Parameter (χ)	10 m/s
Latent Heat of Fusion (*L*_m_)	2.86 × 10^5^ J/kg	Level-Set Parameter (ζ)	1.5 × 10^−5^ m
Latent Heat of Evaporation (*L*_v_)	2.84 × 10^7^ J/kg	Coefficient of Thermal Expansion (*β*_l_)	8 × 10^−4^ K^−1^
Thermal Conductivity (*k*)	30 W/(m/K)	Surface Tension (σ)	1.6–0.00015*T* N/m
Drag Coefficient (K_0_)	6 × 10^4^ kg/(m^3*s)	Surface Tension Gradient (∂σ/∂T)	−0.00015 N/(m/K)
Denominator ($e_{0}$)	0.001	Heat Transfer Coefficient (*h*_c_)	20 W/(m^2^ K^4^)

## Data Availability

The original contributions presented in this study are included in the article. Further inquiries can be directed to the corresponding authors.
